# Neurolocomotor Behavior and Oxidative Stress Markers of Thiazole and Thiazolidinedione Derivatives against *Nauphoeta cinerea*

**DOI:** 10.3390/antiox11020420

**Published:** 2022-02-18

**Authors:** Pedro Silvino Pereira, Adrielle Rodrigues Costa, Thalyta Julyanne Silva de Oliveira, Carlos Vinícius Barros Oliveira, Maria do Carmo Alves de Lima, Jamerson Ferreira de Oliveira, Bonglee Kim, Henrique D. M. Coutinho, Antonia Eliene Duarte, Jean Paul Kamdem, Teresinha Gonçalves da Silva

**Affiliations:** 1 Department of Antibiotics, Federal University of Pernambuco (UFPE), Prof. Artur de Sa Avenue, University City, Recife 54740-520, PE, Brazil; pedro.sillvino@gmail.com (P.S.P.); nenalima.mariadocarmo@gmail.com (M.d.C.A.d.L.); teresinha100@gmail.com (T.G.d.S.); 2 Department of Biological Sciences, Regional University of Cariri (URCA), 1161 Cel. Antonio Luiz Avenue, Pimenta, Crato 63105-000, CE, Brazil; adrielle.arcg@hotmail.com (A.R.C.); julyannebiologia@gmail.com (T.J.S.d.O.); viniciusbluesky@gmail.com (C.V.B.O.); duarte105@yahoo.com.br (A.E.D.); kamdemjeanpaul2005@yahoo.fr (J.P.K.); 3 Institute of Health Sciences, Auroras Campus, University of International Integration of Afro-Brazilian Lusophony (UNILAB), 3 Abolition Avenue, Downtown, Redenção 62790-000, CE, Brazil; jamersonfoliveira@gmail.com; 4 Department of Pathology, College of Korean Medicine, Kyung Hee University, Seoul 02447, Korea; 5 Department of Biological Chemistry, Regional University of Cariri (URCA), 1161 Cel. Antonio Luiz Avenue, Pimenta, Crato 63105-000, CE, Brazil

**Keywords:** toxicity, antioxidant markers, neurotoxic agent, *N. cinerea*

## Abstract

Thiazolidine compounds NJ20 {(E)-2-(2-(5-bromo-2-methoxybenzylidene)hydrazinyl)-4-(4-nitrophenyl)thiazole} and NW05 [(2-(benzo (d) (1,3) dioxol-4-ylmethylene)-N-(4-bromophenyl)-thiosemicarbazone] potentiated the effect of norfloxacin in resistant bacteria; however, there are no reports on their effects on *Nauphoeta cinerea* in the literature. The objective of this work was to evaluate the behavioral effects and oxidative markers of NW05 and NJ20 in lobster cockroach *N. cinerea*. To evaluate the behavioral study, a video tracking software was used to evaluate the locomotor points and the exploratory profile of cockroaches in the horizontal and vertical regions of a new environment. The total concentration of thiol and reduced glutathione (GSH), substances reactive to thiobarbituric acid (TBARS), free iron (II) content and mitochondrial viability were determined. The antioxidant potential was evaluated by the DPPH method. Both substances induced changes in the behavior of cockroaches, showing a significant reduction in the total distance covered and in the speed. In the cell viability test (MTT), there was a significant reduction for NJ20 (1 mM). NJ20 caused a significant increase in total levels of thiol and non-protein thiol (NPSH), although it also slightly increased the content of malondialdehyde (MDA). Both compounds (NW05 and NJ20) caused a significant reduction in the content of free iron at a concentration of 10 mM. In conclusion, the compound NJ20 caused moderate neurotoxicity (1 mM), but had good antioxidant action, while NW05 did not show toxicity or significant antioxidant activity in the model organism tested. It is desirable to carry out complementary tests related to the antioxidant prospection of these same compounds, evaluating them at different concentrations.

## 1. Introduction

The derivatives of thiazolidines, thiazoles and thiazolidinediones are molecules recognized in the literature for presenting a series of relevant pharmacological activities [1,2,3]. Among these activities are antimicrobial [3,4], antiviral [1,2], neurodegenerative [5], antitumor [6,7] and antioxidant [8] activities.

Thiazoles contain nitrogen (N) and sulfur (S) in their heterocyclic rings, which in turn are used as neuroprotectors, playing a leading role in improving antioxidant activity [9,10,11]. This is because they form a metallic complex with considerable biological potential in stabilizing free radicals [12]. Reactive species are known to cause serious damage at the cellular level, such as lipid peroxidation and damage to DNA, proteins and mitochondria, factors directly related to human diseases such as cerebral ischemia, carcinogenesis, diabetes and Alzheimer’s [13,14].

Thiazolidinedione is a heterocyclic ring system with multiple applications. Its nucleus has been reported to be responsible for most of its pharmacological actions. These compounds are known to influence the regulation of different molecular cascades and have several biological activities, such as antimicrobial [4,15], neurodegenerative [5], hypoglycemic [16], antitumor [17], anti-inflammatory [18], and antioxidant [19] activities, among others.

There is evidence in the literature that oxidative stress represents an important mechanism of toxicity of certain substances [20,21,22]. Some substances, under the effect of oxidative stress, can determine low molecular weight thiol depletion, mitochondrial dysfunction, formation of reactive oxygen species (ROSs) and inhibition of the antioxidant enzyme; they can induce undesirable biological reactions, such as lipid peroxidation, protein denaturation and DNA damage, leading to cell death or tissue injury [20,23].

National and international government agencies have defined the need to reduce, refine or replace species of mammals in toxicological tests with alternative testing methods, thus, the lobster cockroach *N. cinerea* is indicated as a promising non-mammalian model in toxicological evaluation and oxidative markers [24,25], as it mimics neurobehavioral and biochemical changes observed in conventional models [24,26]. Since *N. cinerea* shows similarity in the biophysical principles of the nervous system similar to that of mammals [27,28], it can be analyzed for changes in neurotransmitters present in mammals, such as acetylcholine, octopamine and gamma-aminobutyric acid (GABA), and is thus considered a promising model for use in pharmacological, neurobehavioral research and basic toxicological studies [26,29]. In addition, it features easier handling, rapid proliferation/growth and absence of ethical issues compared to rodent-based models.

The use of insects as a model to study the effects of thiazole (NJ20) and thiazolidinedione (NW05) on neurobehavioral changes and oxidative markers is limited, and studies with the lobster cockroach to determine the oxidative stress associated with their use have not been performed. As potential desirable antioxidant drugs in the prevention of oncogenesis, the safety in the use of these compounds should be investigated. Thus, the objective of this research is to evaluate the toxicological effects and oxidative markers of the substances NJ20 and NW05 in the model organism lobster cockroach *N. cinerea*, and indirectly of its neurotoxic effects through the analysis of the locomotor behavior of the exposed animals.

## 2. Materials and Methods

### 2.1. Reagents

Thiazole derivatives NJ20 and NW05 were synthesized by Prof. Dr. Maria do Carmo at the Laboratory of Chemistry and Therapeutic Innovation—LQIT, UFPE [15,30]. All other reagents were purchased from Sigma Aldrich (St. Louis, MO, USA), with analytical grade: glutathione (GSH, PHR1359), 1,1,3,3-tetramethoxypropane (TMP, 108383), 2-Thiobarbituric acid (TBA, T5500), 5,5′-Dithiobis(2-nitrobenzoic acid) (DTNB, D8130), 3-(4,5-Dimethyl-2-thiazolyl)-2,5-diphenyl-2H-tetrazolium bromide (MTT, M5655) and 1,10-phenanthroline (131377).

### 2.2. N. cinerea, Diet Formulation and Treatment

The nymphs of the lobster cockroach *N. cinerea* used in this study were obtained from the Biology and Toxicology Laboratory (BIOTOX), Regional University of Cariri (URCA), Brazil. The animals were reared in plastic boxes with controlled temperature (23–25 °C) and 70% relative humidity, on a 12 h:12 h (light/dark) cycle. Thiazole (NJ20) and thiazolidinedione (NW05) were dissolved in DMSO. Six lobster cockroaches, *N. cinerea*, from each group (thiazole, thiazolidinedione and control) were anesthetized on ice. After this, 10 µL of each compound in concentrations of 1 and 10 mM were administered in the third abdominal segment of three animals each from different groups (six from the thiazole group and six from the thiazolidinedione group), and maintained with free access to food and water in an environment with a temperature of 25 °C for a period of 24 h.

### 2.3. Behavioral Tests for Locomotor Performance

The indirect evaluation of neurotoxic effects can be performed by analyzing the locomotor behavior of animals [31].

### 2.4. Markers of Oxidative Stress

#### Preparation of *N. cinerea* Homogenates for Biochemical Tests

After behavioral analysis, the lobster cockroaches *N. cinerea* were anesthetized on ice, and their heads carefully removed, weighed and manually homogenized in 100 mM potassium phosphate buffer, pH 7.4 (1 mg: 10 μL, weight/volume), and centrifuged at 10,000× *g* rpm for 10 min at 4 °C. The supernatant was separated from the pellet and used to determine mitochondrial viability (MTT reduction), reduced glutathione (GSH), free iron (II) content and lipid peroxidation (TBARS assay). All tests were performed in triplicate in three independent tests, where each test used the supernatant from one animal from each group at the concentrations evaluated.

### 2.5. Assessment of Cell Viability

Cell viability was evaluated with the MTT reagent 3-(4,5-Dimethyl-2-thiazolyl)-2,5-diphenyl-2H-tetrazolium bromide, following the method described by Silva et al. [32].

### 2.6. Measurement of Protein Thiols and Non-Protein Thiol (NPSH) Levels

The levels of protein thiol and non-protein thiol were estimated as an endpoint of oxidative changes in sulfhydryl groups (-SH) of proteins and peptides of the supernatant. Protein thiol and non-protein thiol content were determined by the method described by Ellman [33].

### 2.7. Determination of Species Reactive to 2-Thiobarbituric Acid

2-Thiobarbituric acid reactive substances (TBARS) were measured to determine lipid peroxidation products as a measure of oxidative stress, according to a previous report [34].

### 2.8. Free Fe^2+^ Content Determination

To verify if there was a change in the content of free iron (II) ions in the supernatant of cockroach’s brains, the methodology described previously in the literature was utilized [32,35].

### 2.9. Determination of Antioxidant Activity In Vitro by DPPH Method

The dosage of antioxidant activity was carried out using the photocolorimetric method in vitro, by the sequestration of free radicals DPPH (1,1-diphenyl-2-picrilhidrazil), evaluated according to the method of Kamdem et al. [35]. The radical scavenging activity was measured as a decrease in DPPH absorption, calculated as follows (% inhibition):

[100 − (The sample − A blank)/A control] × 100.(1)

### 2.10. Statistical Analysis

The results were expressed as mean ± standard error of the mean. The experiments were carried out in triplicate in three independent experiments. Statistical differences between groups were performed through analysis of variance (ANOVA), followed by Bonferroni’s test to detect differences between controls and treatments, using the GraphPad Prism^®^ program (version 6) from GraphPad Software 2365 Northside Dr. Suite 560 San Diego, CA, USA. The samples were considered significant with *p* < 0.05.

## 3. Results

### 3.1. Thiazole and Thiazolidinedione—Locomotor Activity

**Figure 1:**
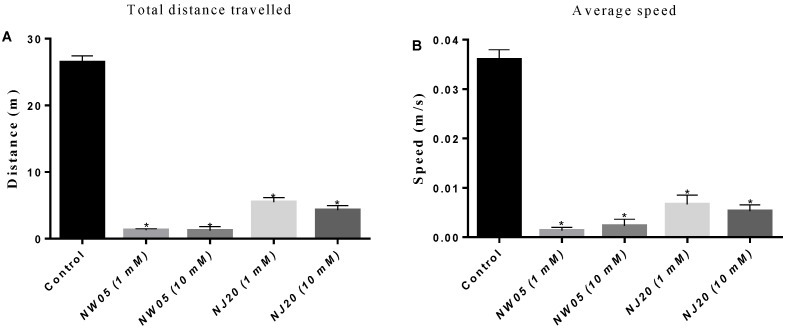

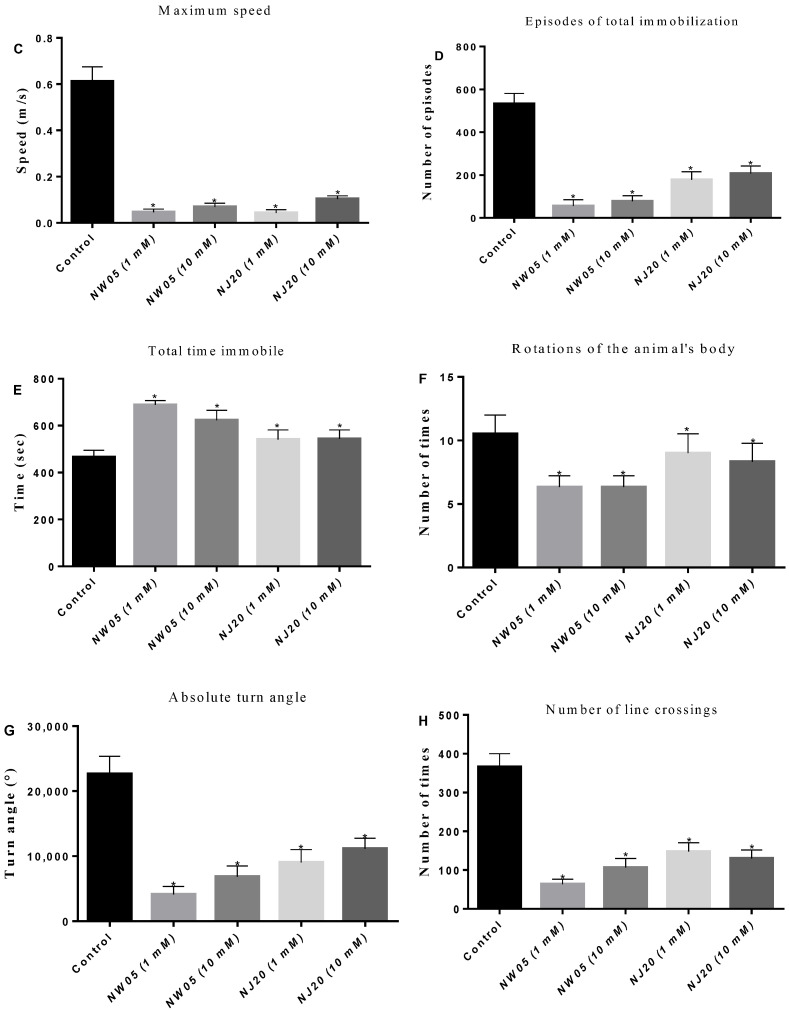
Locomotor performance of lobster cockroaches *N. cinerea* 24 h after treatment with NJ20 and NW05 at concentrations of 1 and 10 mM. (**A**) total distance covered, (**B**) speed, (**C**) Maximum speed, (**D**) episodes of total immobilization, (**E**) total immobile time, (**F**) frequency of rotations, (**G**) absolute turning angle and (**H**) number of line crossings of the lobster cockroaches *N. cinerea* in the open field arena during the 12 min test. The results are averages of at least 5 lobster cockroaches *N. cinerea*. The results were expressed as mean ± standard error of the mean. The experiments were carried out in triplicate in three independent experiments. Statistical differences between groups were performed through analysis of variance (ANOVA), followed by Bonferroni’s test to detect differences between controls and treatments. * Values are statistically different from the control (*p* < 0.05).

To verify the hypothesis that treatment with thiazole derivatives (NJ20) and thiazolidinedione (NW05) may induce changes in the behavior of lobster cockroaches *N. cinerea*, the routine end points of locomotor activity were analyzed during a 12-min study. As shown in Figure 1, cockroaches that were treated with substances NW05 and NJ20 showed a significant decrease for both tested concentrations (1 and 10 mM) in the total distance covered (Figure 1A), average speed (Figure 1B), maximum speed (Figure 1C), total moving time (Figure 1D), rotations of the animal’s body (Figure 1F), absolute angle (Figure 1G) and number of crossings (Figure 1H) when compared with the control group (*p* < 0.05), while the total immobile time (Figure 1E) showed a significant increase for both tested substances. In general, the greatest behavioral variations were caused by the compound NW05, especially at the concentration of 1 mM.

**Figure 2:**
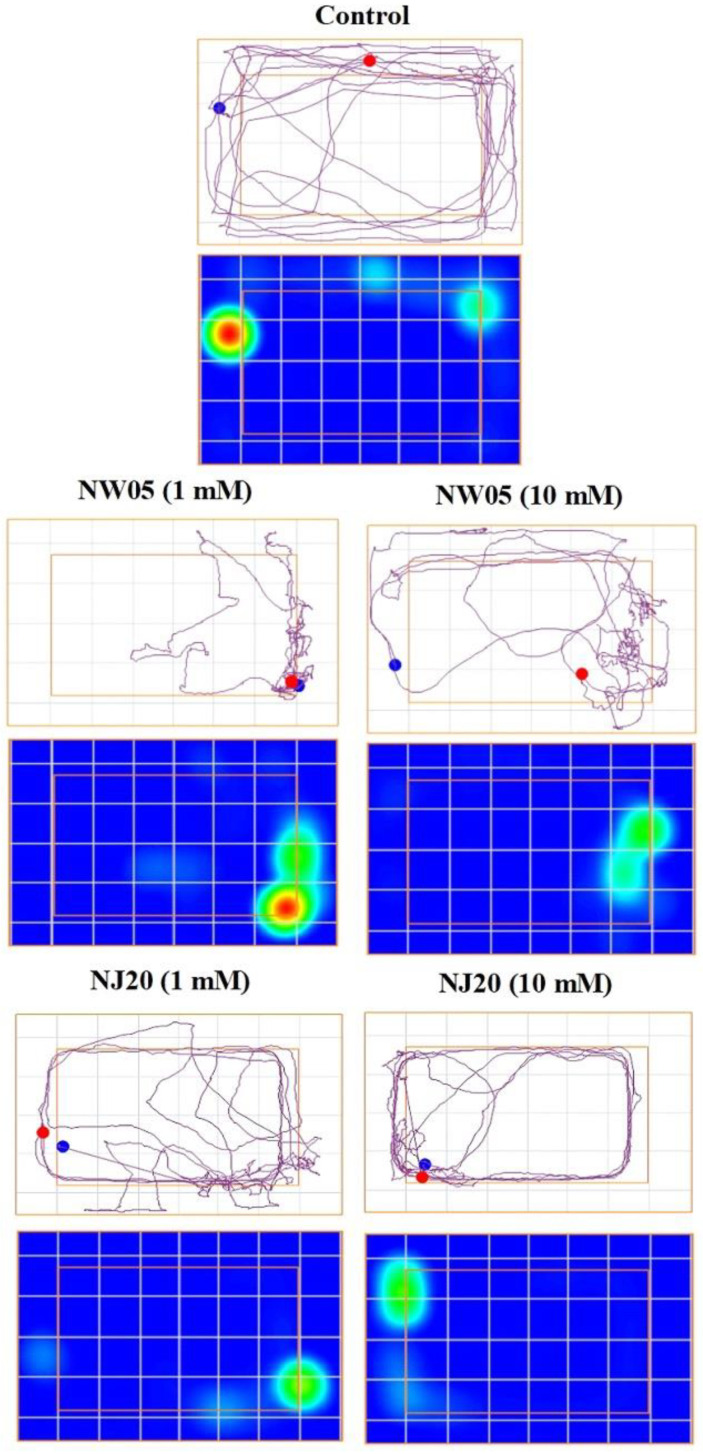
Representative track plots of the path travelled and heat map by lobster cockroaches *N. cinerea* (24 h after treatment with NJ20 and NW05 at concentrations of 1 and 10 mM) in the open-field arena during the 12 min trial. The smaller-area orange square indicates the boundaries of the container’s bottom, while the larger orange square indicates the container’s edges. In the heat maps, cells are about 4 cm^2^.

Consistently, the representative portions of the trails that show the path taken by the cockroaches of the control group and administered with NW05 and NJ20 indicate that the control group had greater locomotor activity when compared to the treated groups (Figure 2). The heat map of the lobster cockroaches *N. cinerea* specimens represented in Figure 2 shows in plots the colors in which the *N. cinerea* cockroaches roamed; the light green plot indicates the decrease in heat of the specimens, since they did not remain immovable in the environment, and when they remained still, the color turns red, thus showing a greater heat in the place when they remain immobile. Therefore, control group and NW05 treated group (1 mM) had red plots indicating, thus, immobile time of *N. cinerea* specimens.

### 3.2. Cells Viability

**Figure 3:**
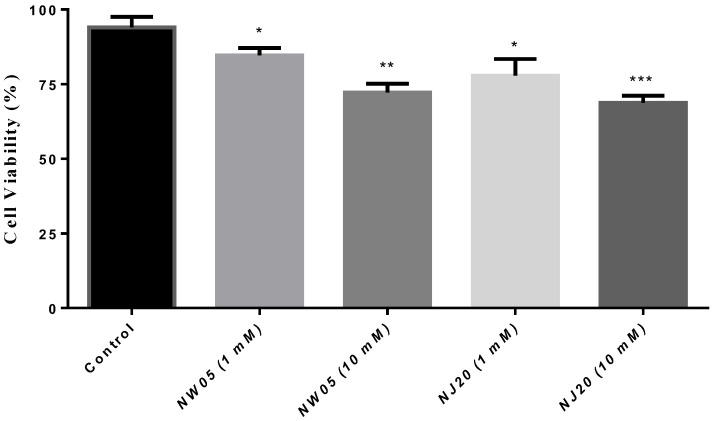
Effect on cell viability in lobster cockroaches *N. cinerea* treated with thiazole (NJ20) and thiazolidinedione (NW05). The results were expressed as mean ± standard error of the mean. The experiments were carried out in triplicate in three independent experiments. Statistical differences between groups were performed through analysis of variance (ANOVA), followed by Bonferroni’s test to detect differences between controls and treatments. * The values are statistically different from the control (*p* < 0.05), ** (*p* < 0.01), *** *p* < 0.001.

The 3-(4,5-dimethylthiazolyl-2)-2,5-diphenyltetrazolium (MTT) cell proliferation assay was used to measure cell viability. This assay is performed with MTT dye that is reduced by the action of the dehydrogenase enzymes present in the mitochondria of viable cells for purple formazan. Figure 3 shows that cell exposure to thiazole NJ20 at a concentration of 10 mM caused a significant reduction in mitochondrial activity, consequently affecting cell viability.

### 3.3. Measurement of Protein and Non-Protein Thiols (NPSH)

**Figure 4:**
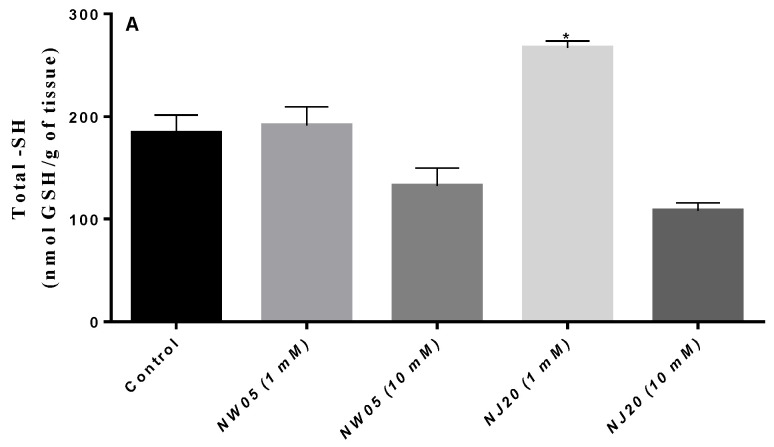

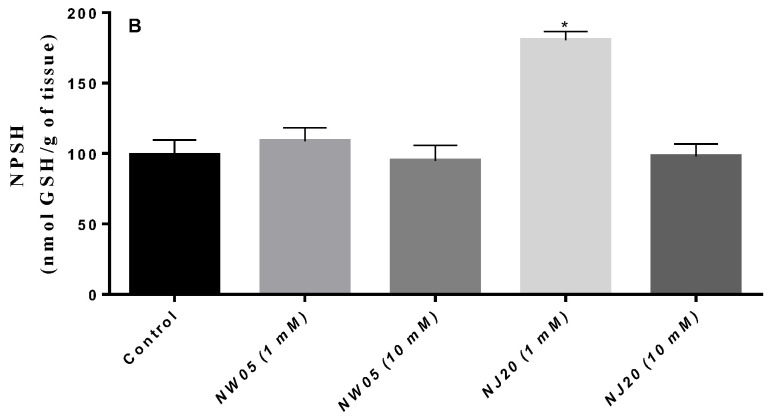
Total levels of proteins (**A**) and non-protein thiols (GSH) (**B**) in lobster cockroaches *N. cinerea* treated with thiazole (NJ20) and thiazolidinedione (NW05). The results were expressed as mean ± standard error of the mean. The experiments were carried out in triplicate in three independent experiments. Statistical differences between groups were performed through analysis of variance (ANOVA), followed by Bonferroni’s test to detect differences between controls and treatments. * The values are statistically different from the control (*p* < 0.05).

Analyzing the levels of protein (Figure 4A) and non-protein thiols (Figure 4B), it is possible to observe a depletion in the levels of thiols (nmol GSH/g of tissues) in the highest concentration (10 mM) of the two substances tested when compared to the control group, as shown in Figure 4. A variation is observed in the levels of the control group and of individuals treated with NW05 and NJ20 in the concentrations (1 mM and 10 mM), except for NJ20 (1 mM), where the levels had a statistically significant increase for both protein and non-protein thiols.

### 3.4. Measurement of Malondialdehyde (MDA) Content

**Figure 5:**
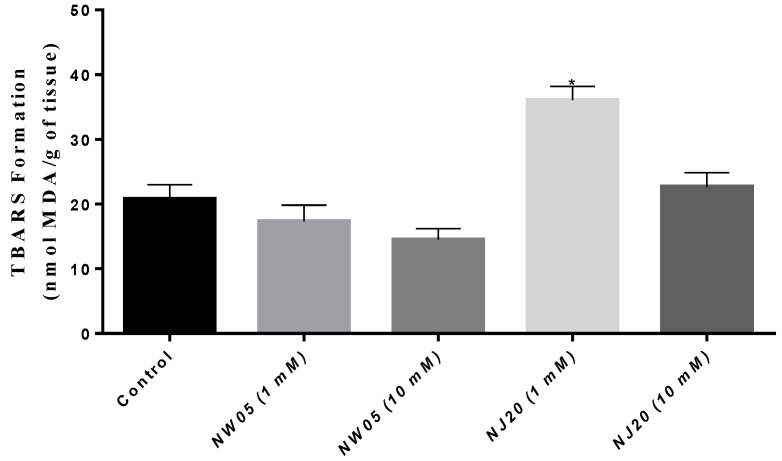
Malondialdehyde (MDA) content in *N. cinerea* treated with thiazole (NJ20) and thiazolidinedione (NW05). The results were expressed as mean ± standard error of the mean. The experiments were carried out in triplicate in three independent experiments. Statistical differences between groups were performed through analysis of variance (ANOVA), followed by Bonferroni’s test to detect differences between controls and treatments. * The values are statistically different from the control (*p* < 0.05).

The results of lipid peroxidation are represented by the levels of malondialdehyde (MDA) as a final product of lipid degradation by measuring the formation of substances reactive to thiobarbituric acid (TBARS). As shown in Figure 5, NW05 showed a reduction in MDA levels, while NJ20 showed a significant increase in MDA levels, mainly in the concentration of 1 mM, compared to the control group (*p* < 0.05), demonstrating a high index of lipid peroxidation caused by the exposure of this substance. However, cockroaches treated with relatively higher concentrations (10 mM) did not show a significant increase in MDA content when compared to the control group.

### 3.5. Determination of Total Free Iron Levels

**Figure 6:**
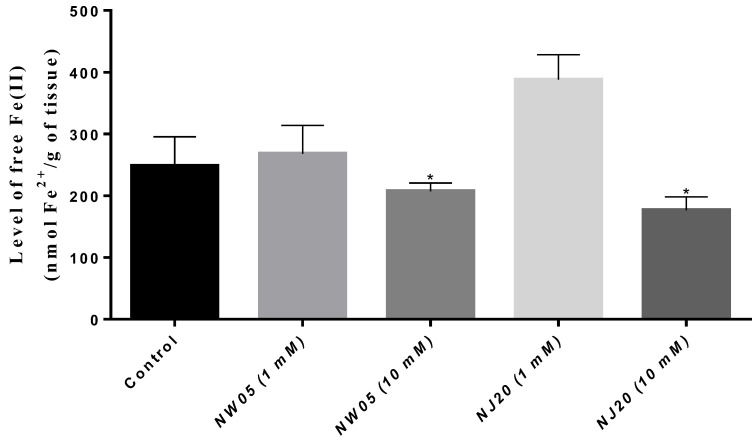
Free iron level in lobster cockroaches *N. cinerea* homogenates treated or not with thiazole (NJ20) and thiazolidinedione (NW05). The results were expressed as mean ± standard error of the mean. The experiments were carried out in triplicate in three independent experiments. Statistical differences between groups were performed through analysis of variance (ANOVA), followed by Bonferroni’s test to detect differences between controls and treatments. * The values are statistically different from the control (*p* < 0.05).

The free Fe^2+^ ion content was determined by measuring the intensity of the orange complex formed by the interaction of free Fe^2+^ plus 1.10-phenanthroline in the supernatant. Free Fe^2+^ ions are known to play an essential role in vital cell functions at relatively lower concentrations; however, high levels are indicative of toxicity. As shown in Figure 6, in the treatment with NW05 and NJ20 it was possible to observe a significant reduction in the total iron content in the concentration of 10 mM in comparison with the control group (*p* < 0.05). However, a substantial increase in the total iron content was observed with NJ20 at a concentration of 1 mM.

### 3.6. Evaluation of Antioxidants by the DPPH Test

**Figure 7:**
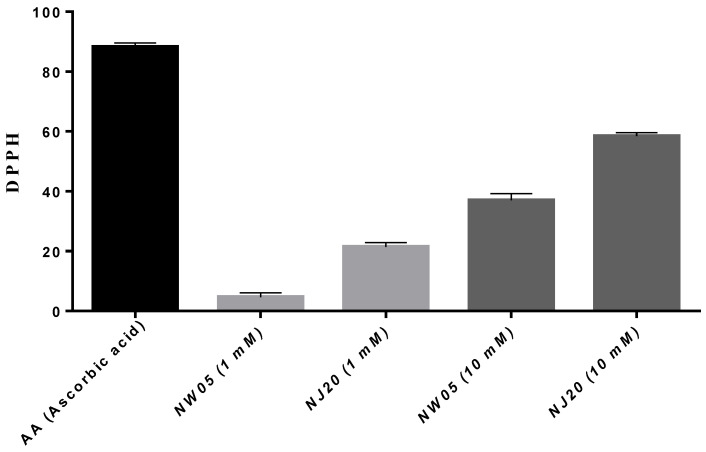
Antioxidant effect of thiazole (NJ20) and thiazolidinedione (NW05). The results were expressed as mean ± standard error of the mean. The experiments were carried out in triplicate in three independent experiments. Statistical differences between groups were performed through analysis of variance (ANOVA), followed by Bonferroni’s test to detect differences between controls and treatments.

Antioxidants protect cells against the damaging effects of free radicals which result in oxidative stress, leading to cell damage. It can be seen in Figure 7 that ascorbic acid (used as a reference substance) has considerable antioxidant property when compared to NW05, which in both concentrations (1 mM) and (10 mM) showed less antioxidant capacity, and NJ20 in both concentrations (1 mM) and (10 mM) showed greater antioxidant capacity when compared to NW05 (Figure 7).

## 4. Discussion

The use of alternative models to assess the toxic effects and safety of chemical substances has been widely encouraged, and in this context, the lobster cockroach *N. cinerea* proved to be a promising model for conventional tests. Adedara et al. [24,36] point out that the use of the species *N. cinerea* in research is relevant to outline toxicological and behavioral mechanisms. In the present study, the behavioral evaluation of cockroaches and changes in biochemical parameters induced by oxidative stress was analyzed after exposure to substances NW05 and NJ20, similarly to that performed by countless other highly relevant studies carried out in recent years [35,37,38], such as the study by Rodrigues et al. [38] when evaluating oxidative stress induced by mercury in *N. cinerea*, Waczuk et al. [39] analyzing the toxic effect of 4-vinylcyclohexane in nymphs of *N. cinerea* and Adedara et al. [40] evaluating the impact of exposure to diclofenac on the behavior and antioxidant defense system of *N. cinerea*.

Analyzing our results, it is possible to observe a change in the behavioral parameters of the animals tested with both substances (NJ20 and NW05), showing a significant reduction in terms of total distance covered, both in general aspects such as agility and speed and in body rotation and turn angle (Figure 1A–H). These results may be associated with the inhibition of sensorimotor coordination induced by the use of substances, affecting the animal’s body movements [41] and probably interfering with the cholinergic neurotransmission of cockroaches [26,32]. In *N. cinerea*, aspects related to body agility are inversely proportional to the size of the specimen [41]. No studies were found that tested thiazolidine derivatives in a *N. cinerea* models; however, thiazole compounds can cause significant behavioral changes in vertebrates (zebrafish) by the ability to bind to γ-aminobutyric acid (GABA) receptors [42]. Wang et al. [43], when evaluating the neuroprotective effect and mechanism of thiazolidinedione in dopaminergic neurons in mice treated with 2,4-thiazolidinedione, found changes in motor functions.

The heat map of *N. cinerea*, represented in Figure 2, depicts its exploratory behavior. The control group and the group treated with NW05 (1 mM) presented plots in red, indicating, therefore, that the specimens remained immobilized for a long time. Exploratory behavior is important for the survival of specimens in their habitat, resulting from the fact that full exploration of the environment helps to obtain food and escape predators [40].

According to Silva et al. [32], oxidative stress is a condition characterized by an imbalance between the body’s antioxidant defense and reactive species, which can often be induced by common exogenous substances, where a possible antioxidant homeostasis is triggered by the deleterious action of using certain drugs, often inducing cytotoxicity, causing significant variations in cell viability, which can be evaluated by the MTT reduction assay [44]. The results of the cell viability assessment test performed on specimens treated with compounds NW05 and NJ20 indicated that thiazole (NJ20) caused a significant increase in cytotoxicity (1 mM), evidenced by the reduction in cell viability (Figure 3), an effect observed for several other thiazole derivatives tested on insects [45,46].

Certain substances with toxic properties can have serious effects on the levels of cellular markers, demonstrating an increase of the immediate protective response, for example, the intensification of the transcription levels of genes encoding antioxidant molecules [47], such as protein thiol, which are reducing agents in the stability of free radicals [36,37], and which suffer depletion in the cytotoxicity framework [48]. Our results showed that at the lowest concentrations of NW05 (1 mM) and NJ20 (1 mM) there was an increase in the levels of protein thiols in the homogenates of *N. cinerea*, thus indicating the antioxidant capacity of the tested compounds (Figure 4A). When studying the action of diphenyl diselenide (DPDS), a compound widely reported for exhibiting antioxidant and neuroprotective effects from in vitro and in vivo studies in various biochemical mechanisms, increased levels of protein thiols were observed using a concentration of 20 µmol in a protein thiol test in a Drosophila model organism melanogaster [37]. Therefore, compounds used even at low concentrations may have antioxidant activity, as observed in our results.

As for antioxidant markers such as thiols that are natural antioxidants, glutathione (GSH), a non-protein thiol and an important tripeptide in the redox system, has a significant role in the homeostatic balance of the cell [32,49,50,51,52]. The levels of non-protein unions in *N. cinerea* homogenates at NW05 (1 mM) and NJ20 (1 mM) concentrations were high when compared to the control group (Figure 4B). In the study by Rodrigues et al. [38] evaluating the toxicity of mercury, a reduction in the levels of protein thiols was observed. The same author justifies that the oxidative stress induced by mercury is attributed to the formation of ROS via depletion of thiols with low molecular mass [24].

Lipid peroxidation (LPO) is characterized as irreversible degradation of plasma membrane lipids, generating a series of degradation products such as malondialdehyde (MDA); its significant levels are detectable as species reactive to thiobarbituric acid (TBARS), a marker of oxidative stress [53]. The substance NJ20 caused a significant increase in MDA at a concentration of 1 mM (Figure 5), similar to the effect observed for sodium glutamate, a potent neurotoxic agent [49,54]. However, NW05 caused a reduction in levels when compared to the control group, results that corroborate those of Bittencourt et al. [55], who found that isolated caffeine decreased the damage induced by lipid peroxidation by 25%.

Oxidative markers such as excess free Fe^2+^ are considered indicative of toxicity at the molecular level. According to some researchers, a high level of free Fe^2+^ ions and GSH have been detected in several diseases, including cancer [56,57,58]. In this context, substances capable of chelating free iron in order to reduce its availability in the environment are of great interest [59]. In relation to our results of free iron levels, a significant reduction was observed in connective tissue homogenates of *N. cinerea* after treatment with NW05 (10 mM) e NJ20 (10 mM) (Figure 6). It has been reported that certain thiazoles have excellent free Fe^2+^ chelating capacity and moderate toxicity, which could justify the results found here [60].

Substances with antioxidant potential of both natural and synthetic origin are being studied due to their prophylactic potential in the therapeutic capacity of various diseases [61,62]. The DPPH assay indicates the ability to sequester the DPPH radical, in which it transfers electrons or hydrogen atoms to the DPPH radical, stabilizing the test radical [63]. In vitro, the substance must exhibit this behavior in the organism as well. In our results, NW05 (1 mM and 10 mM) showed less antioxidant capacity, while NJ20 (1 mM and 10 mM) showed higher antioxidant capacity when compared to NW05 (1 mM and 10 mM) (Figure 7). When studying combretina A and B in mice, Marius et al. [64] found the antioxidant capacity of the compounds, but ascorbic acid stood out in terms of antioxidant activity.

Thiazoles have a broad spectrum of biological activity, including good antioxidant activity, as demonstrated by Silva et al. [65], due to the dissipation of thiazole ring radicals [66], justifying the study of these important nuclei. Likewise, thiazolidine-2,4-dione derivatives are extensively studied for their exceptional antioxidant properties [67]. Heterocyclic compounds containing the thiazolidine portion play an important role in the field of medicinal chemistry, presenting a wide range of biological activities, from antihypertensive to simple anti-inflammatory activity [68].

## 5. Conclusions

The compounds NW05 and NJ20 decreased the locomotor capacity of *N. cinerea* in all assays; only NJ20 (1 mM) caused a significant increase in the levels of protein and non-protein thiols, which may indicate the activation of compensatory mechanisms to inhibit its toxicity, although it also increases the content of MDA and reduces cell viability. Both compounds (NW05 and NJ20) limit the chelating capacity of Fe^2+^ to a concentration of 10 mM. From the observation, it can be said that the results of the compounds in the tests vary according to their millimolarity, and it is desirable to carry out complementary tests related to the antioxidant prospection of these same compounds, especially related to their chelating capacity, evaluating them at different concentrations. Furthermore, the present work highlighted *N. cinerea* as a viable model for toxicological assessments—not only biochemical, but also behavioral.

## Data Availability

Data is contained within the article.
